# CULD is required for rhodopsin and TRPL channel endocytic trafficking and survival of photoreceptor cells

**DOI:** 10.1242/jcs.178764

**Published:** 2016-01-15

**Authors:** Ying Xu, Tao Wang

**Affiliations:** 1 School of Life Sciences, Beijing Normal University, Beijing, China, 100875; 2 National Institute of Biological Sciences, Beijing, China, 102206

**Keywords:** TRPL, Rhodopsin, Endocytosis, Phototransduction, CULD

## Abstract

Endocytosis of G-protein-coupled receptors (GPCRs) and associated channels contributes to desensitization and adaptation of a variety of signaling cascades. In *Drosophila melanogaster*, the main light-sensing rhodopsin (Rh1; encoded by *ninaE*) and the downstream ion channel, transient receptor potential like (TRPL), are endocytosed in response to light, but the mechanism is unclear. By using an RNA-Sequencing (RNA-Seq) approach, we discovered a protein we named CULD, a photoreceptor-cell enriched CUB- and LDLa-domain transmembrane protein, that is required for endocytic trafficking of Rh1 and TRPL. CULD localized to endocytic Rh1-positive or TRPL-positive vesicles. Mutations in *culd* resulted in the accumulation of Rh1 and TRPL within endocytic vesicles, and disrupted the regular turnover of endocytic Rh1 and TRPL. In addition, loss of CULD induced light- and age-dependent retinal degeneration, and reduced levels of Rh1, but not of TRPL, suppressed retinal degeneration in *culd*-null mutant flies. Our data demonstrate that CULD plays an important role in the endocytic turnover of Rh1 and TRPL, and suggest that CULD-dependent rhodopsin endocytic trafficking is required for maintaining photoreceptor integrity.

## Abstract (precis)

**Summary:** CULD, a CUB- and LDLa-domain transmembrane protein that is enriched in photoreceptor cells, is required in endocytic turnover of rhodopsin and TRPL.

## INTRODUCTION

G-protein-coupled receptors (GPCRs) are the largest family of membrane receptors and, therefore, transduce signals from a wide variety of hormones, cytokines, neurotransmitters, as well as sensory stimuli. Each of these interactions triggers distinct intracellular responses through heterotrimeric G proteins (Katritch et al., 2013). Upon continuous stimulation, GPCRs are deactivated by arrestins, and internalized through dynamin-dependent endocytosis. Many internalized GPCRs undergo lysosomal degradation and/or recycling, leading to downregulation of receptor levels, which is important for reducing the strength and duration of cellular responsiveness following various stimuli (Bunnett and Cottrell, 2010; Drake et al., 2006; Hanyaloglu and von Zastrow, 2008).

The *Drosophila* phototransduction cascade is a model pathway for the dissection of GPCR signaling and associated regulatory processes (Griciuc et al., 2012; Montell, 2012; Wang and Montell, 2007). Proteins of the visual signal transduction cascade are found within rhabdomeres, which are specialized compartments within photoreceptor cells that contain tightly packed microvilli. Light-induced activation of rhodopsin triggers the phototransduction cascade by stimulating the vision protein phospholipase C, which is encoded by the no receptor potential A (*norpA*) gene, through the α subunit of the heterotrimeric G protein DGq (Lee et al., 1990). This opens the transient receptor potential (TRP) channel and the TRP-like (TRPL) Ca^2+^/cation channel, and depolarizes the photoreceptor neurons (Montell, 2012; Wang and Montell, 2007). Meanwhile, activated rhodopsin, which is referred to as metarhodopsin, is immediately bound by arrestin and deactivated. After inactivation, metarhodopsin is either photoconverted back into rhodopsin or internalized for degradation. Although the majority of internalized metarhodopsin is degraded, with newly synthesized rhodopsin replenishing the pool, it has recently been reported that internalized rhodopsin (Rh1; encoded by *ninaE* in *Drosophila melanogaster*) can be recycled upon stimulation with light (Satoh and Ready, 2005; Wang et al., 2014, 2010; Xiong et al., 2012; Xiong and Bellen, 2013). The principle arrestin, Arr2, plays a pivotal role in deactivating rhodopsin, whereas Arr1 binds and internalizes rhodopsin (Satoh and Ready, 2005).

Long-term adaptation to light stimuli also involves the dynamic activity-dependent translocation of signaling proteins that are not GPCRs. As seen with mammalian Rod photoreceptors, light induces the movement of Arr2 and Arr1 into the rhabdomeres (Calvert et al., 2006; Lee et al., 2003; Satoh and Ready, 2005), and Gαq and TRPL out of the rhabdomeres (Bähner et al., 2002; Kosloff et al., 2003). In *Drosophila*, TRP and TRPL function as the primary light-activated channels (Minke and Parnas, 2006; Wang and Montell, 2007). TRP stably localizes to the rhabdomeres by forming a multiprotein signaling complex, the signalplex with inactivation-no-after-potential D protein (INAD), a protein that contains five PDZ domains (Tsunoda et al., 1997). In contrast, illumination results in TRPL translocating from the rhabdomeres to an intracellular storage compartment within the cell body (Bähner et al., 2002). However, the mechanisms that underlie light-induced translocation and trafficking of rhodopsin and TRPL are not yet fully understood. Furthermore, it is unclear whether this endocytic trafficking of TRPL plays a physiological role in maintaining the integrity of photoreceptor cells.

By using an RNA-Sequencing (RNA-Seq) approach, we identified a so-far-unknown gene that is enriched in photoreceptors, and encodes a transmembrane protein with both a CUB and an LDLa domain. We named this protein CULD (CUB- and LDLa-domain protein). We found that CULD mainly localized to the endocytic TRPL- or Rh1-positive vesicles. Mutations in *culd* led to endosomal accumulation of Rh1 and TRPL, which disrupted the light sensitivity of photoreceptors; blocking of Arr1-mediated endocytosis eliminated the intracellular accumulation of Rh1. Moreover, *culd* mutants underwent light-dependent retinal degeneration, and resulted in a phenotype that could be rescued by reducing the levels of Rh1. Our data indicate that CULD is essential for the function and survival of photoreceptor cells by promoting the endocytic turnover of Rh1 and TRPL.

## RESULTS

### CULD is predominantly expressed in photoreceptor cells

A previous study used microarray analysis to compare the genes expressed in heads of wild-type *Drosophila* with those from a mutant fly that lacked eyes, in order to identify eye-enriched genes (Xu et al., 2004). Because there are several different cell types in the compound eye, many genes identified this way are not involved in photoreception. Moreover, some photoreceptor-enriched genes are likely to have been missed. To identify new genes that are required for photoreceptor physiology and survival we conducted an RNA-Seq screen for genes that are predominantly expressed in photoreceptors. The *glass* (*gl*) gene encodes a zinc finger transcription factor, and *gl* mutations specifically remove photoreceptor cells but leave other cell types intact (Moses et al., 1989). In the heads of *gl^3^* flies, Rh1, which is expressed exclusively in photoreceptor cells, was completely absent. In contrast, levels of the retinal pigment cell marker photoreceptor dehydrogenase (PDH) were unchanged (Wang et al., 2010).

**Fig. 1.**
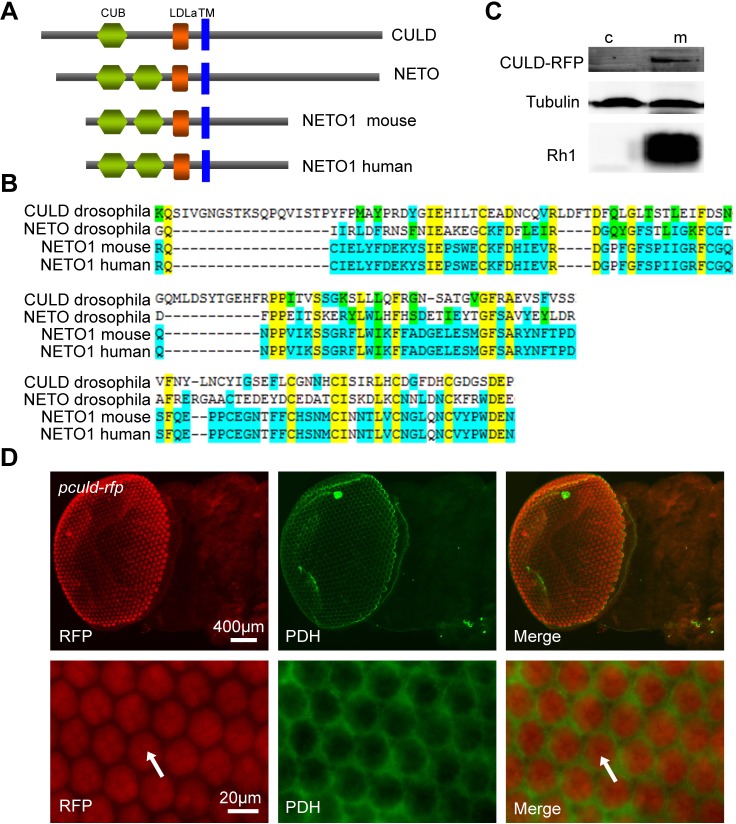
**CULD is a new photoreceptor-enriched CUB/LDLa-domain family protein.** (A) Domain organization of CULD. Indicated are the CUB domain(s), LDLa domain and predicted transmembrane motif (TM) of CULD, *Drosophila* NETO, mouse NETO1 and human NETO1. The domains were predicated by InterPro (http://www.ebi.ac.uk/InterProScan/). (B) The alignment of the CUB domain and LDLa domain among *Drosophila* NETO, mouse NETO1, and human NETO1. The top two rows are the alignment of CUB domain, and the alignment of LDLa domains is on the bottom. Yellow shows residues conserved across all proteins, blue shows residues conserved across some of the proteins and green shows similar residues. (C) CULD is a transmembrane protein. The cytoplasmic (c) and membranal (m) fractions of the *ninaE-culd-rfp* head extracts were separated and western blots are probed with anti-tubulin, anti-Rh1 and anti-RFP antibodies. Extracts from four heads were loaded for each lane. (D) Expression of *culd* is enriched in photoreceptor cells. Heads from *Pculd-rfp* flies at 70% pupal development express RFP (red) driven by the *culd* promoter are shown. Eye tissue was stained with anti-PDH (green) and anti-RFP (red) antibodies. The photoreceptor cells are indicated by arrows.

By comparing mRNAs isolated from wild-type (*w^1118^*) heads with *gl*^3^ heads or wild-type bodies, we identified 58 genes that were expressed predominantly in photoreceptor cells. Among these 58 genes, 36 were known to function in photoreceptor cells, representing most of the genes that play major roles in adult photoreceptors (Table S1). A total of 22 of the genes had not been described as enriched in photoreceptor cells (Table 1), including *cg17352*, which encodes a transmembrane protein containing a CUB (C1r/C1s, Uegf, Bmp1) domain and a low-density lipoprotein class A (LDLa) domain (Fig. 1A,C), which we thus name CULD (CUB- and LDLa- domain). As multiple attempts to generate an antibody against CULD failed, we instead analyzed *culd* promoter activity to confirm the expression of CULD in photoreceptor cells. We generated a transgenic fly that expressed red fluorescent protein (RFP) under the control of the *culd* promoter (*Pculd-rfp*). RFP localized exclusively to photoreceptor cells in retina, and the signals did not overlap with PDH, which is expressed in pigment cells (Fig. 1D) (Wang et al., 2010). These results confirm that the *culd* gene encodes a protein that is enriched in photoreceptor cells.

**Table 1.**
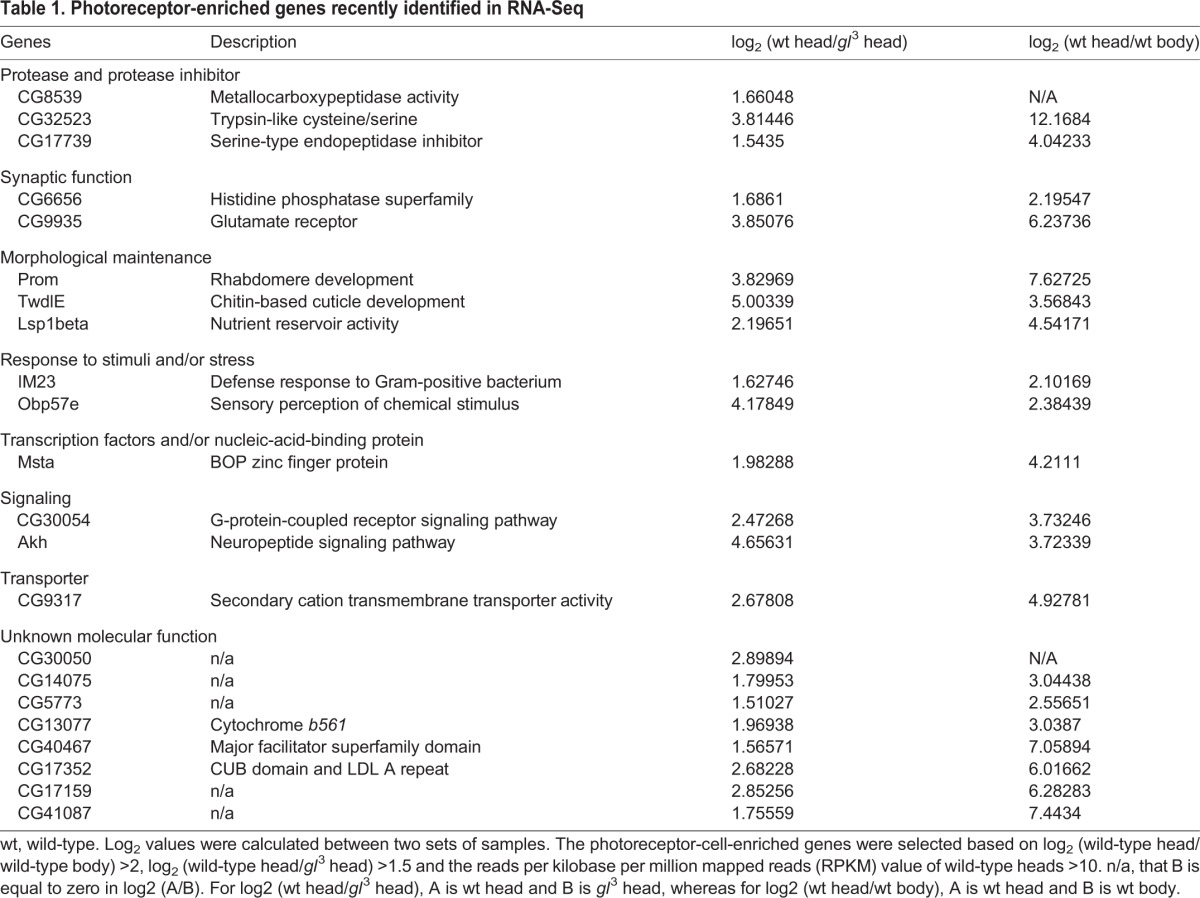
**Photoreceptor-enriched genes recently identified in RNA-Seq**

A group of CUB/LDLa-domain family proteins has previously been identified in vertebrates and invertebrates (for a selection, see Fig. 1A,B). Among these proteins, neuropilin and tolloid-like-1 and -2 (NETO1 and NETO2, respectively) are required for the clustering and functioning of glutamate receptors (Kim et al., 2012; Ng et al., 2009; Zhang et al., 2009). We, therefore, reasoned that the photoreceptor-enriched protein CULD might be required for correct function of the light receptor rhodopsin, and/or the downstream ion channels TRP and TRPL.

### CULD is required for normal physiology of Rh1 and TRPL

**Fig. 2.**
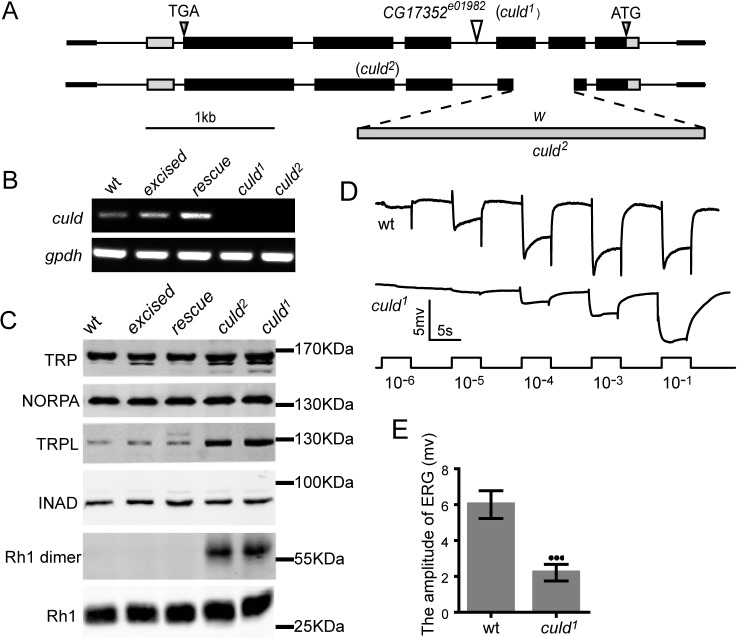
**Mutations in *culd* increase the protein levels of TRPL and Rh1 and decrease light sensitivity.** (A) Organization of the *culd* locus and two *culd* alleles. The *culd^1^* allele consists of a PiggyBac insertion in the third intron of *culd*. The *culd^2^* allele lacks most of the second and third exons, a deletion induced by gene targeting. (B) Both *culd^1^* and *culd^2^* mutations disrupt *culd* transcription. *gpdh* served as a loading control. The genotypes are as follows: wild-type (wt), *w^1118^*; *excised*, a precise excision of *culd^1^*; *rescue*, *ninaE-culd-rfp, culd^1^*. (C) Western blotting revealed that *culd* loss-of-function increased TRPL levels and the amount of Rh1 aggregations. Protein extracts from half a head were loaded for each lane. Four-day-old flies raised under 12-h-light–12-h-dark cycles were used. (D) ERG recordings from 4-day-old wt (*cn bw*) and *culd^1^* (*cn bw*; *culd^1^*) flies. Flies were dark-adapted for 2 min before exposed to five pulses of white light of increasing intensities. The maximal light intensity is 300 Lux (10^−1^). (E) Quantification of the ERG amplitudes of wt and *culd^1^* flies in the intensity of 0.3 Lux (10^−4^ in D). Error bars represent s.d. (*n*=7). ***P<0.001 (unpaired *t*-test).

To characterize the role of CULD in the physiology of photoreceptor cells, we generated mutations within the *culd* locus. We first obtained the PiggyBac insertion line *cg17352^e01982^* (*culd^1^*), which contains an insertion within the third intron of the *culd* gene. From this, we generated a null mutation in the *culd* locus (*culd^2^*) by using homologous recombination (Fig. 2A). Both mutants are null alleles of *culd* because the *culd* mRNA was totally absent (Fig. 2B).

As the mammalian CULD homologues NETO1 and NETO2 regulate the abundance of associated receptors and transporters (Ivakine et al., 2013; Tang et al., 2011), we asked whether CULD regulates the protein levels of Rh1, TRP, TRPL or other components of the phototransduction pathway. Western blot analysis of *culd^1^* and *culd^2^* flies revealed increased levels of TRPL, but normal levels of TRP, NORPA and INAD (Fig. 2C). Although levels of the monomeric form of Rh1 did not increase significantly, Rh1 tended to form aggregates of high molecular mass in *culd^1^* and *culd^2^* mutants (Fig. 2C). To verify that these changes in TRPL and Rh1 resulted from *culd* loss-of-function, we restored CULD in the *culd^1^* mutant by precise excision of the PiggyBac element (excised) or by expressing an RFP-tagged version of CULD from the *ninaE* (*rh1*) promoter (*ninaE-culd-rfp, culd^1^*: rescue), which drives gene expression in the R1–R6 photoreceptor cells. In both ‘excised’ and ‘rescue’ flies TRPL levels were reduced to wild-type levels, and Rh1 aggregates were eliminated (Fig. 2C). These data suggest that CULD is required for Rh1 and TRPL homeostasis.

To test whether CULD regulates the visual responses, we analyzed vision responses of *culd^1^* flies by performing electroretinogram (ERG) recordings, which measure the summed response of all retinal cells. The ERG response of young *culd^1^* flies was normal, indicating that CULD did not play a direct role in the phototransduction cascade. However, aged *culd^1^* mutants (4-day-old) exhibited dramatically decreased light sensitivity (Fig. 2D,E). Importantly, this reduction in light sensitivity did not result from the loss of photoreceptor cells, because photoreceptors were intact in 4-day-old *culd^1^* mutants.

### Rh1 and TRPL accumulate in intracellular vesicles of *culd^1^* photoreceptors

**Fig. 3.**
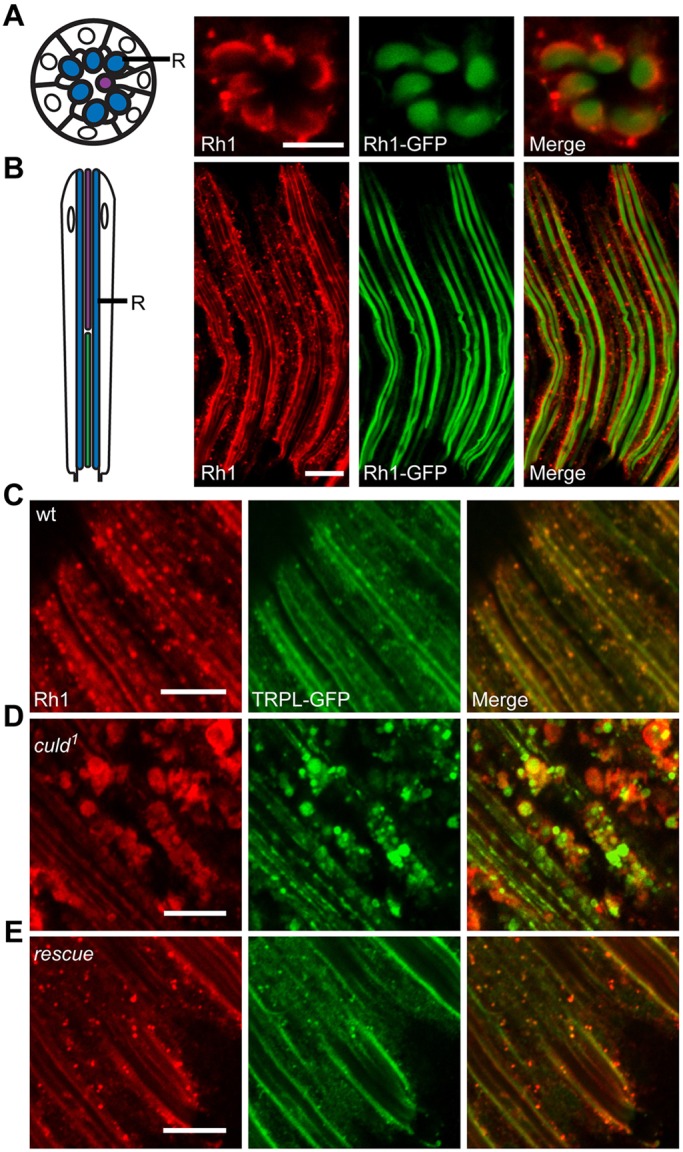
**Rh1 and TRPL accumulate within *culd^1^* photoreceptors.** (A,B) The left row shows a schematic diagrams of cross-sectional (A) and longitudinal views (B) of photoreceptor cells from a single ommatidium. The rhabdomere (R) is indicated. The right rows show the whole-mount staining of Rh1 in cross-sectional (A) and longitudinal (B) views. Eyes from *ninaE-rh1-gfp* flies were dissected and stained for Rh1 (red). GFP fluorescence of Rh1–GFP was directly observed (green). (C–E) Rh1 and TRPL accumulated in large vesicles within the cytoplasm of *culd^1^* photoreceptor cells. Compound eyes from (C) wild-type (wt) (*ninaE-trpl-gfp*), (D) *culd^1^* (*cn bw; ninaE-trpl-gfp culd^1^*) and (E) rescue (*cn bw; ninaE-culd-rfp culd^1^*) flies were dissected and immunostained for Rh1. The *ninaE-trpl-gfp* transgene was present in all genotypes and GFP signals were directly observed. Two-day-old flies with white eyes were placed in the dark for 12 h before being exposed to 2000 Lux white light for 2.5 h. Scale bars: 10 µm.

The compound eye of the fly consists of ∼800 ommatidia; each ommatidium has eight photoreceptor cells, including a microvillar structure, the rhabdomere (Fig. 3A,B). We found that Rh1 was stained at the base of rhabdomeres in a streaky pattern, which was probably due to the limited access of antibodies to densely organized microvillar structures in whole-mount samples (Chinchore et al., 2009). GFP-tagged Rh1 was uniformly distributed throughout the rhabdomere, which could be observed directly, i.e. without staining, whereas the endocytic Rh1 could only be visualized following antibody staining (to amplify the signals) (Fig. 3A,B). Because of the length of photoreceptor cells, longitudinal views of photoreceptors are more representative for the endocytic vesicles than cross-section views and we, therefore, only show longitudinal views of the samples. In wild-type retinas that had been exposed to light, most Rh1 was found in the base of rhabdomeres, although some were found in regular and small cytosolic vesicles (Fig. 3C). In *culd^1^* flies, however, Rh1 was accumulated within a number of enlarged cytosolic vesicles (Fig. 3D). Owing to lack of good antibodies for TRPL staining, GFP-tagged TRPL was used to check the accumulation of TRPL in *culd^1^* flies (Meyer et al., 2006). After a 2.5-h light treatment, some TRPL translocated into the cytosol and was found in many regular and small vesicles within wild-type flies. Moreover, TRPL also accumulated within the enlarged cytosolic vesicles in *culd^1^* flies, and a substantial amount of Rh1 and TRPL accumulated in the same vesicles (Fig. 3D). These phenotypes could be rescued completely by expressing RFP-tagged CULD in photoreceptor cells from the *ninaE* promoter (*ninaE-culd-rfp, culd^1^*) (Fig. 3E).

### The block of Arr1-mediated endocytosis eliminates accumulation of Rh1 in *culd^1^* flies

**Fig. 4.**
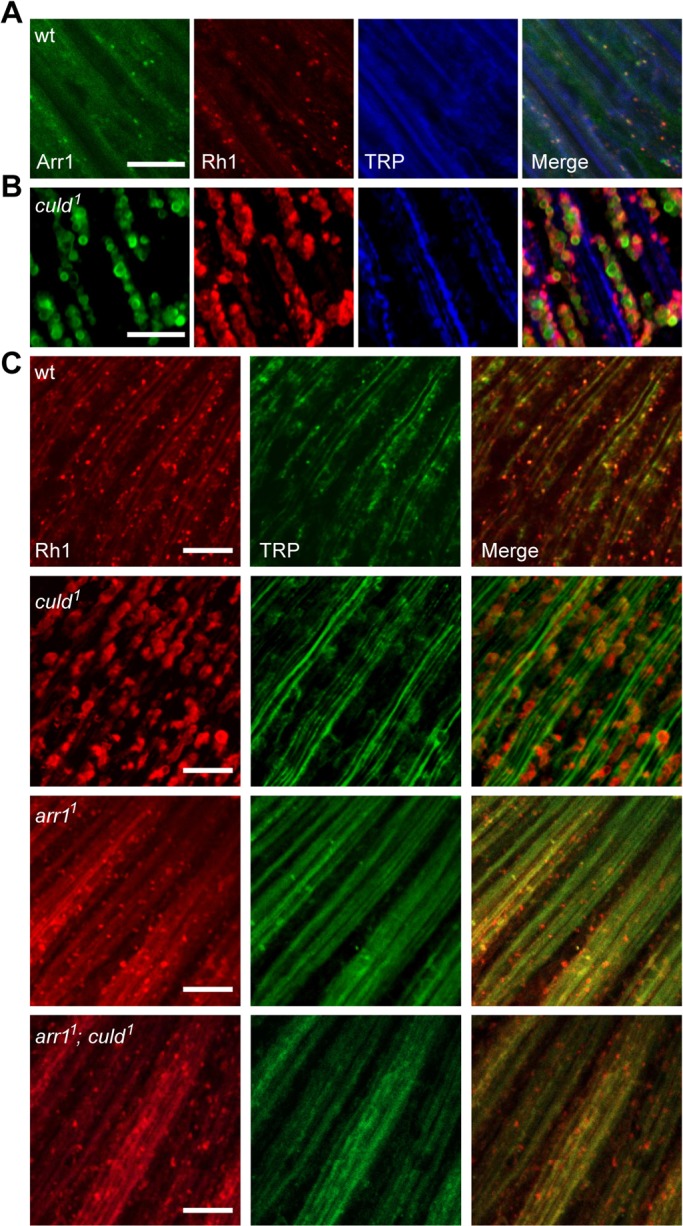
**Mutation of *arr1* blocked the intracellular accumulation of Rh1 in *culd^1^*.** (A,B) Rh1 colocalized with Arr1 in *culd^1^* flies. Eyes from *cn bw; ninaE-arr1-bfp* (wild-type, wt) and *cn bw; ninaE-arr1-bfp, culd^1^* (*culd^1^*) flies were dissected and stained for Rh1 (red) and TRP (blue). BFP fluorescence of Arr1–BFP was directly observed (green). (C) Retinas of wt (*w^1118^*), *culd^1^* (*cn bw; culd^1^*), *arr1^1^* (*arr1^1^ cn bw*), and *arr1^1^; culd^1^* (*arr1^1^ cn bw; culd^1^*) flies were dissected and immunostained for Rh1 (red) and TRP (green). Two-day-old flies with white eyes raised under 12-h-light–12-h-dark cycles were used and placed in the dark for 12 h, then exposed to 2000 Lux white light for 7 h. Scale bars: 10 µm.

Degradation or sequestration of GPCRs following agonist stimulation typically occurs through arrestin-dependent internalization (Claing et al., 2002). *Drosophila* photoreceptors express two arrestins, Arr1 and Arr2. Upon light exposure, Arr1 and Arr2 translocate to the rhabdomere from the cell body. Arr2 quenches activated rhodopsin, Arr1 localizes to endocytic Rh1 and is essential for normal, light-induced Rh1 endocytosis (Satoh and Ready, 2005). Therefore, we wondered whether vesicular accumulation of Rh1 in *culd* mutant photoreceptor cells was epistatic to Arr1-mediated endocytosis. To test this hypothesis, we first generated transgenic flies that expressed Arr1–BFP from the *ninaE* promoter (*ninaE-arr1-bfp*). After 7 h of exposure to light, almost all Arr1 was detected in enlarged Rh1-positive vesicles within *culd^1^* photoreceptor cells, whereas in wild-type retinas, some Arr1 was found in the cytoplasm, with a certain proportion in endocytic Rh1-positive vesicles (Fig. 4A,B). We next checked the intracellular accumulation of Rh1 in *arr1^1^; culd^1^* double-mutant flies, and found that loss of Arr1 rescued the accumulation of large Rh1-positive vesicles in *culd^1^* mutants (Fig. 4C). These results suggest that CULD functions downstream of Arr1-mediated endocytosis of Rh1.

### CULD colocalized with endocytic Rh1 and TRPL

**Fig. 5.**
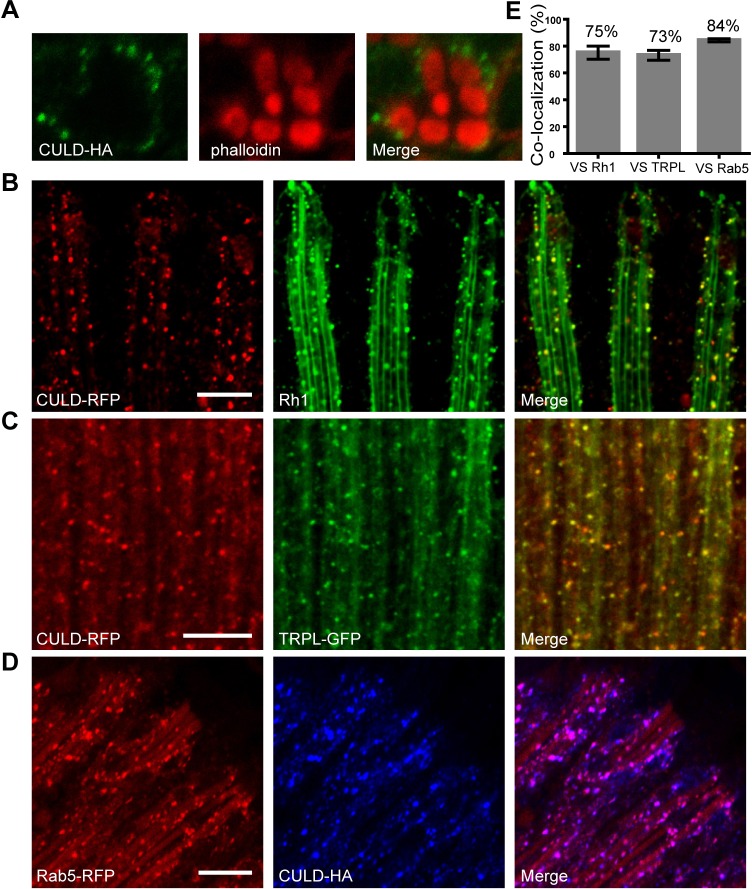
**CULD locates in TRPL- or Rh1-positive endocytic vesicles in photoreceptor cells.** (A) Cross-sectional images of retina dissected from *ninaE-culd-HA* (*cn bw; ninaE-culd-HA*) flies were stained with anti-HA antibody for detecting CULD (green) and with phalloidin for labeling the rhabdomere (red). (B) Longitudinal images of retina dissected from *cn bw; ninaE- culd-rfp/+* flies were labeled with anti-Rh1 antibody for detecting Rh1 (green) and with anti-RFP for detecting CULD (red). (C) Retinas of *cn bw; ninaE-culd-rfp/ninaE-trpl-gfp* flies were stained with anti-RFP (red for CULD) and anti-GFP (green for TRPL) antibodies. (D) Retinas of *cn bw; ninaE-culd-HA/ninaE-rab5-rfp* flies were labeled with anti- HA (blue for CULD) and anti-RFP (red for RFP) antibodies. Two-day-old flies with white eyes were placed in the dark for 12 h before exposed to 2000 Lux white light for 2.5 h. Scale bars: 10 µm. (E) Quantification of the colocalization between CULD and the endocytic Rh1, TRPL and Rab5. Number of vesicles positive for CULD and/or Rh1, TRPL or Rab5 were counted in confocal sections as described in Materials and Methods, and divided by the total number of CULD-positive vesicles. Images of retinas from three different flies and at least five 20 µm×20 µm areas of each retina were quantified. Error bars represent s.d.

To further address the function of CULD, we first examined the subcellular localization of CULD through transgenic flies that express RFP- or HA-tagged CULD proteins in photoreceptor cells from the *ninaE* promoter (*ninaE-culd-rfp* and *ninaE*-*culd-HA*), which is able to rescue the *culd^1^* phenotype. By staining retina tissues, we found that CULD was distributed in intracellular vesicles within photoreceptor cells, which were exclusively outside of rhabdomeres (Fig. 5A). In *Drosophila* photoreceptors, during illumination, TRPL and Rh1 translocate from the rhabdomeres to cell bodies, using the same vesicular transport pathway (Oberegelsbacher et al., 2011). We, therefore, asked whether CULD localized to endocytic TRPL- or Rh1-positive vesicles. After 2.5 h of illumination with white light, a lot of TRPL- and Rh1-positive vesicles were observed in a longitudinal view of photoreceptors. A large proportion of CULD-positive vesicles was Rh1-positive or TRPL-positive (75% or 73%, respectively; Fig. 5B,C,E). The early endosomal protein Rab5 is an important mediator of vesicular transport pathways involved in TRPL and Rh1 translocation, and Rab5 was found in most endocytic particles containing Rh1 and TRPL (Han et al., 2007). We, therefore, checked whether CULD colocalized with RFP-tagged Rab5 and found a strong colocalization between CULD and Rab5 (84%). These results indicate that CULD is required in early endosomes to maintain the normal endocytosis of Rh1 and TRPL (Fig. 5D,E).

### CULD is required for endocytic turnover of Rh1 and TRPL

**Fig. 6.**
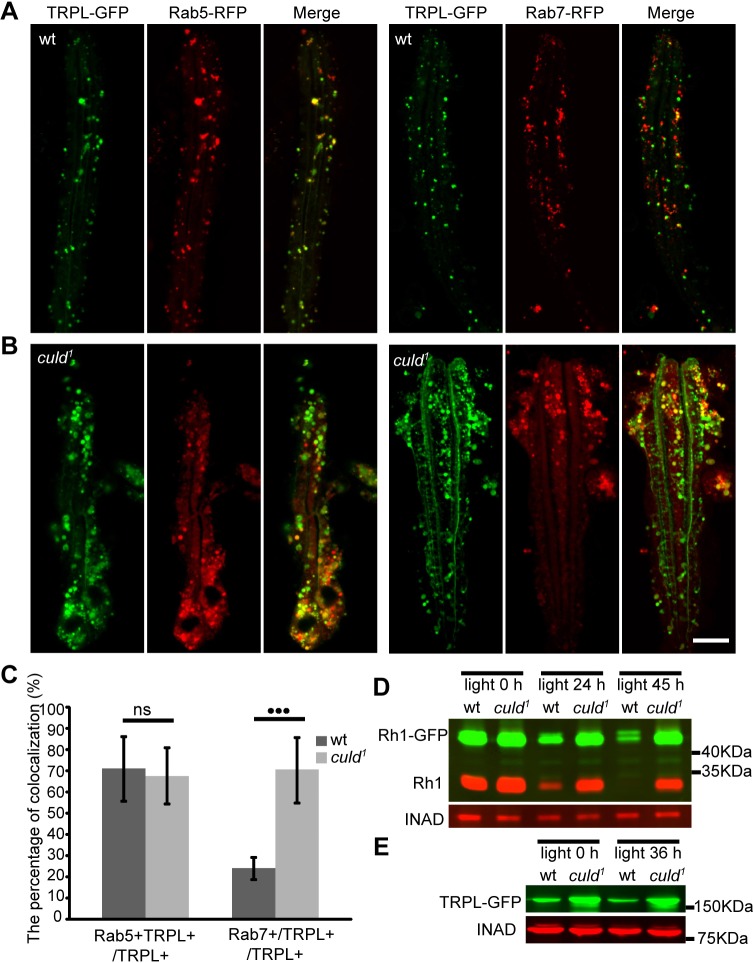
**Defective light-dependent turnover of Rh1 and TRPL in *culd^1^* flies.** (A,B) Colocalization between (A) Rab5–RFP (red) and (B) Rab7–RFP with TRPL–GFP (green) in ommatidia. Three-day-old flies with *ninaE-trpl-gfp* and *ninaE-rab5-rfp* or *ninaE-rab7-rfp* transgenes in either wild-type or *culd^1^* background were placed in the dark for 12 h, before exposed to orange light for 2 h. Scale bar: 10 µm. (C) Quantification of the percentage of TRPL and Rab5 double-positive vesicles among TRPL-positive vesicles (TRPL+Rab5+/TRPL+) and the percentage of TRPL and Rab7 double-positive vesicles among TRPL-positive vesicles (TRPL+Rab7+/TRPL+) are shown for the experiments in A and B. At least nine ommatidia were quantified for each sample as described in Materials and Methods. Error bars indicate s.d. ***P<0.001; ns, not significant (unpaired *t*-test). (D) Light-induced Rh1 degradation was blocked in *culd^1^* flies. Western blots of heads were from *ninaE-rh1-gfp; cn bw; culd^1^*/TM6B (wt) and *ninaE-rh1-gfp; cn bw; culd^1^* (*culd^1^*) flies exposed to blue light for the indicated periods of time. INAD served as a loading control, and protein extracts from half of a fly head were loaded for each line. (E) Western blot analysis of heads from *ninaE-trpl-gfp* (wt) or *cn bw; ninaE-trpl-gfp, culd^1^* (*culd^1^*) flies that were exposed to orange light for indicated periods of time. Protein extracts of one head were loaded for each line. Two-day-old wt or *culd^1^* flies with white eyes were used.

The subcellular localization of CULD suggests that it functions in endocytic trafficking of Rh1 and TRPL. We used RFP-tagged Rab proteins and GFP-tagged TRPL (TRPL–GFP) to monitor the endocytic trafficking of TRPL in *culd^1^* ommatidia. Rab5 is an early endosomal marker and has been found in most endocytic particles that contain Rh1 and TRPL; Rab7 is commonly used as a marker for late endosomes. The percentages of Rab5-positive vesicles among the endocytic TRPL were similar between wild-type and *culd^1^* flies after a 2-h treatment with orange light (Fig. 6A,C). However, a significantly higher percentage of endocytic TRPL vesicles were Rab7-positive in *culd^1^* retinas compared with wild-type retinas (Fig. 6B,C). Therefore, the endocytic trafficking of TRPL might be blocked in late endosomes in *culd* mutants. This blockage of TRPL trafficking might disrupt degradation of TRPL and contribute to the increased protein levels of TRPL that is observed in *culd* mutants.

It has been suggested that endocytic Rh1 is degraded in a lysosome-dependent pathway (Xu et al., 2004) and a lot of endocytic Rh1 was blocked in the same vesicles together with TRPL in *culd^1^* flies. We, therefore, asked whether loss of CULD blocks the endocytic degradation of Rh1. Two-day-old flies were exposed to blue light for 24 h or 45 h, which converts Rh1 into the light-activated metarhodopsin and triggers Rh1 endocytic degradation. In wild-type animals, Rh1 levels were dramatically lower after the 24-h treatment with blue light (Fig. 6D). However, reductions in Rh1–GFP and Rh1 in response to blue light were largely blocked in *culd^1^* mutants (Fig. 6D). We also treated 2-day-old flies with orange light to monitor TRPL homeostasis because stimulation with orange light results in maximum levels of TRPL internalization (Oberegelsbacher et al., 2011). TRPL protein levels barely decreased in response to 36 h of orange light in both wild-type and *culd^1^* flies (Fig. 6E). This result is consistent with a previous report, describing that TRPL levels remain constant despite the light-induced endocytosis of TRPL because most of TRPL protein is stored in a storage compartment after endocytosis (Bähner et al., 2002). These data suggest that CULD participates in the turnover of Rh1 and TRPL by promoting their normal endocytic trafficking.

### Mutations in *culd* lead to age- and light-dependent retinal degeneration

**Fig. 7.**
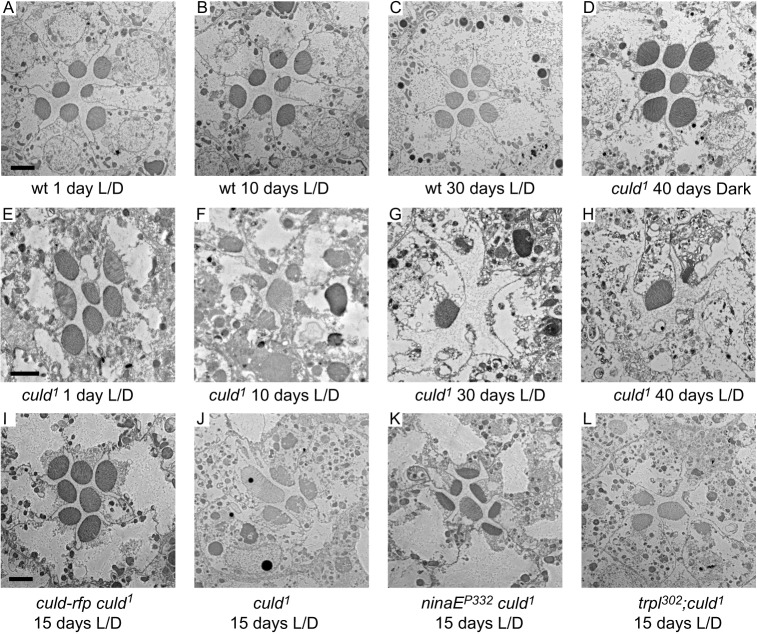
**Light-dependent retinal degeneration in *culd^1^* flies.** Ommatidial morphologies are shown by TEM examinations. Sections were obtained from (A–C) wt (*w^1118^*) or (D–H) *culd^1^* (*cn bw;culd^1^*) flies, which were maintained under 12-h-light–12-h-dark (L/D) cycles or in the dark for indicated periods of time. (I–L) Suppression of retinal degeneration in *culd^1^* flies by *ninaE^P332^*. (I) *ninaE-culd-rfp,culd^1^*, (J) *culd^1^*, (K) *ninaE^P332^,culd^1^*, and (L) *trpl^302^; culd^1^* flies were raised for 15 days under 12-h-light–12-h-dark cycles. The light intensity is 2000 Lux. Scale bars: 2 µm.

To determine whether the integrity of photoreceptors depends on CULD, we assayed the death of photoreceptor cells in *culd^1^* ommatidia by using transmission electron microscopy (TEM). In tangential sections of the compound eye, a single ommatidium includes seven photoreceptor cells with centrally located rhabdomeres. We found that *culd^1^* flies exhibited late-onset and light-dependent retinal degeneration. In wild-type ommatidia, seven rhabdomeres were detected regardless of ages and light conditions (Fig. 7A–C). However, *culd^1^* flies reared in a 12-h-light–12-h-dark cycle exhibited gradual losses of rhabdomeres and photoreceptor cells (Fig. 7E–H). After 10 days in the light–dark cycle, the number and size of *culd^1^* rhabdomeres were reduced (Fig. 7F). Images with high magnification showed that abnormal vesicles were accumulated in *culd^1^* flies during degeneration process, and these vesicles resembled the mutivesicular bodies (Fig. S1). At the age of 30 days, *culd^1^* photoreceptor cells were severely degenerated and there was a complete loss of rhabdomeres of R1–R6 cells (Fig. 7G). However, R7 and R8 photoreceptor cells were still intact (Fig. 7G), because different rhodopsins are expressed in R7 and R8 cells (Wang and Montell, 2007). All seven rhabdomeres were present in dark-reared *culd^1^* flies at the age of 40 days (Fig. 7D,H), and the light sensitivity of old *culd* mutant flies is also maintained in the dark condition (Fig. S2). These results are consistent with less endocytosis of Rh1 and TRPL in dark conditions than that in light conditions. Moreover, the degeneration of photoreceptors in *culd^1^* mutants was completely rescued by expressing CULD–RFP (*ninaE-culd-rfp, culd^1^*) (Fig. 7I).

To determine whether the degradation block of TRPL and/or Rh1 was the main cause of retinal degeneration in *culd^1^* flies, we eliminated Rh1 or TRPL in the *culd^1^* mutant background. The *trpl^302^; culd^1^* flies displayed severe retinal degeneration at the age of 15 days under light–dark cycles (comparable to *culd^1^*; Fig. 7L,J). In contrast, ommatidia of *ninaE^P332^, culd^1^* double mutant flies had seven rhabdomeres, which were small because of reduced Rh1 levels in *ninaE^P332^* flies (Fig. 7K). These results demonstrate that the retinal degeneration in *culd* mutants is primarily caused by the defective endocytic turnover of Rh1.

## DISCUSSION

In this study we showed that a new CUB/LDLa-domain family protein, CULD, is required for the light-dependent turnover of rhodopsin and TRPL. As such, CULD maintained the integrity of photoreceptor cells. Loss of CULD resulted in the intracellular accumulation of Rh1 and TRPL within large endosomal vesicles, leading to disruptions of the degradation of rhodopsin, which caused the degeneration of photoreceptor cells.

### Photoreceptor-enriched genes in flies

A microarray analysis has previously been used to compare the genes expressed in wild-type heads with heads from a mutant fly that lacked eyes in order to identify eye-enriched genes, which led to the further identification of some genes functioning in phototransduction (Xu et al., 2004). However, owing to multiple cell types in the compound eye, many genes identified in this analysis might not function in photoreceptor cells. Here, we conducted an RNA-Seq screen to identify genes expressed predominantly in photoreceptors. Among the 58 genes identified, 36 genes were known to function in photoreceptor cells, representing most of the genes that play major roles in phototransduction or retinal degeneration. However, 22 genes had not been described as being enriched in photoreceptor cells previously. Among them, *cg9935* (*ekar*) has been recently reported to regulate the retrograde glutamate signal in photoreceptor cells and contribute to light-evoked depolarization during phototransduction (Hu et al., 2015). We further characterized the new photoreceptor cell-enriched gene *culd* as being required for turnover of Rh1 and TRPL. Although *culd* had also been identified as an eye-enriched gene in the earlier microarray analysis that compared RNA expression in wild-type and *eyeless* heads, 93 other eye-enriched candidates prevented us from focusing on CULD (Xu et al., 2004). In this RNA-Seq screen, only photoreceptor-cell-enriched genes can be identified, and a reasonable number of candidates might represent new factors functioning in phototransduction. However, some eye-enriched genes important for phototransduction might be missed in this screen. For example, recently identified polyglutamine-binding protein 1 (*PQBP1*) was not found as a photoreceptor-cell-specific gene in our RNA-Seq screen (Wan et al., 2015). This might be because *PQBP1* is also expressed in other non-photoreceptor retinal cells. Overall, this screen for photoreceptor-enriched genes sheds a light on further understanding of phototransduction and mechanisms of retinal degeneration.

### CULD functions in endocytic trafficking pathways of Rh1 and TRPL

Appropriate signals cause arrestins to translocate to the plasma membrane where they bind to activated GPCRs, thereby inhibiting G-protein-dependent signaling and regulating GPCR endocytosis and trafficking (Shenoy and Lefkowitz, 2011). In *Drosophila* there are two arrestins within photoreceptors, Arr1 and Arr2. Although Arr2 binds to Rh1, it is Arr1 that primarily colocalizes with Rh1 in internalized vesicles. Therefore, Arr1 might mediate light-dependent endocytosis of Rh1, whereas Arr2 functions to quench activated Rh1 (Dolph et al., 1993; Satoh and Ready, 2005). In *culd* mutant flies, Rh1 was immobilized within endocytic vesicles and Arr1 colocalized with the endocytic Rh1; blocking the Arr1-medicated endocytosis in *culd* mutant cells eliminated the abnormal intracellular accumulation of Rh1. These data strongly suggest that CULD functions downstream of Arr1-mediated endocytosis of Rh1.

Early endosomes containing Rab5 serve as a focal point of the endocytic pathway. Sorting events initiated in early endosomes determine the subsequent fate of internalized proteins, that is, whether they will be recycled to the plasma membrane or degraded within lysosomes (Kelly and Owen, 2011; Mayor et al., 1993). Rh1 and TRPL share the same internalization pathway, and during light stimulation Rab5 initially mediates this vesicular transport pathway (Han et al., 2007; Oberegelsbacher et al., 2011). In wild-type photoreceptors, however, Rh1 and TRPL have different fates from common Rab5-positive early endosomes. The majority of Rh1 is eventually delivered to lysosomes for degradation, whereas most internalized TRPL tends to be stored (Bähner et al., 2002; Wang et al., 2014). In wild-type cells, the photoreceptor-enriched protein CULD colocalized with the endocytic TRPL or Rh1 vesicles, and the majority of CULD-positive vesicles were also Rab5-positive. This spatial pattern indicates that CULD is required for the endocytic trafficking of TRPL and Rh1 after they are internalized.

### CULD is required for maintaining the normal photoreceptor physiology

CULD functions during the early steps of endocytosis that immediately follow internalization, which is a pathway involved in rhodopsin and TRPL endocytic turnover. Eliminating CULD had profound effects on the photoreceptor physiology. In both vertebrates and invertebrates, the light sensitivity of photoreceptor cells is primarily determined by functional rhodopsin (Wang and Montell, 2007). The *culd* mutant flies exhibited a gradual reduction in light sensitivity, which suggests that the amount of functional rhodopsin is reduced in *culd* mutant flies. As the amount of the monomer form of Rh1 was not affected and a large fraction of Rh1 accumulated within intracellular vesicles in *culd* mutant photoreceptor cells, the rhabdomeral Rh1 levels might be reduced. It is also likely that the endocytic degradation of Rh1 scavenges damaged Rh1 molecules, and blocking this process might lead to the accumulation of dysfunctional Rh1 in rhabdomeres.

### Turnover of TRPL and rhodopsin

TRPL has been reported to translocate from rhabdomeres to intracellular compartments for storage during prolonged light stimulation. However, a recent study suggests that some endocytic TRPL proteins are also delivered to lysosomes for degradation (Cerny et al., 2013). Mutations in *culd* impaired TRPL endocytic trafficking upon light stimulation, leading to the retention of TRPL in Rab7-positive vesicles. We found that TRPL protein levels were increased in *culd* mutants and this is probably due to decreased TRPL degradation.

As a major light sensor within photoreceptor cells, a small amount of activated Rh1 is internalized and degraded upon light stimulation. This is followed by replenishment of the rhabdomeric Rh1 pool. Therefore, a balance between Rh1 endocytosis and replenishment is required for Rh1 homeostasis under light conditions. Prolonged exposure to blue light triggers massive endocytosis of Rh1 and leads to a gradual loss of Rh1. Mutations in *culd* blocked Rh1 degradation during prolonged light treatment, indicating that the loss of CULD inhibited the Rh1-degradation pathway. Unlike TRPL, Rh1 levels were not increased, which suggests that Rh1 replenishment is strictly controlled. Given that, in *Drosophila*, rhodopsin levels are regulated by both the synthesis of the opsin and the chromophore subunits, it might be reasonable that in *culd* mutant cells, the chromophore is not released from the accumulated rhodopsin, and the reduction of free retinal pool might limit the synthesis of new rhodopsin.

### The role of CUB- and LDLa-domain proteins

Both vertebrates and invertebrates have a family of transmembrane proteins that contain both CUB- and LDLa- domains (Bork and Beckmann, 1993; Christensen and Birn, 2002). However, only a few CUB/LDLa proteins have been functionally characterized. Among these proteins, NETO1 and NETO2 have been intensively studied (Stöhr et al., 2002). NETO1 functions as an auxiliary subunit of ionotropic glutamate receptors, N-methyl-D-aspartate receptors and kainate receptors, modulating the channel properties of these glutamate receptors (Ng et al., 2009; Straub et al., 2011; Tang et al., 2011; Zhang et al., 2009). NETO2 maintains normal levels of the neuron-specific K^+^–Cl^−^ co-transporter KCC2 (also known as SLC12A5), and loss of NETO2–KCC2 interactions reduces KCC2-mediated Cl^−^ extrusion, and decreases synaptic inhibition in hippocampal neurons (Ivakine et al., 2013). Moreover, in both *Drosophila* and *C. elegans*, the CUB/LDLa proteins NETO and SOL-2 are required for the clustering and functioning of glutamine receptors, thereby contributing to neuronal signaling pathways (Kim et al., 2012; Wang et al., 2012). Here, we cloned a new gene *culd*, which encodes a member of the CUB/LDLa family proteins specifically expressed in the *Drosophila* photoreceptor cell; this protein containing a CUB domain, an LDLa domain and one predicted transmembrane motif. CULD was not directly required for the activity of receptors or channels, but instead mediated the endocytic trafficking of Rh1 and TRPL. Loss of CULD led to the accumulation of Rh1 and TRPL in endocytic vesicles, and subsequent retinal degeneration. Therefore, our study revealed a new function of the CUB/LDLa family proteins, namely the endocytic turnover of receptors and channels.

### Retinal degeneration caused by defective rhodopsin turnover

It has been proposed that the accumulation of Rh1–Arr2 complexes in late endosomes triggers cell death of photoreceptor cells. Internalized Rh1–Arr2 complexes are not degraded but instead accumulate in late endosomes of *norpA* mutant photoreceptor cells. Similar rhodopsin accumulations are seen in mutations that affect the trafficking of late endosomes to lysosomes, which causes light-dependent retinal degeneration (Chinchore et al., 2009). Toxic Rh1–Arr2 complexes also induce retinal degeneration in *rdgC*, *rdgB* and *fatp* mutant flies (Alloway et al., 2000; Dourlen et al., 2012; Kiselev et al., 2000). The *culd* mutations caused the accumulation of Rh1–Arr1 complexes and TRPL in endocytic vesicles and light-dependent retinal degeneration, suggesting that endosomal accumulation of either channels or receptors induced cell death. In addition, the evidence that *ninaE^P332^* but not *trpl^302^* rescued photoreceptor degeneration of the *culd^1^* mutants suggests that abnormal Rh1–Arr1 accumulation induces cell degeneration, whereas intracellular accumulation of TRPL does not contribute to the neuronal degeneration.

## METHODS AND MATERIALS

### Fly stocks

The following stocks were obtained from the Bloomington Stock Center: (1) *y w; P[70FLP]11 P[70I-SceI]2B nocSco/CyO* used for generating the *culd^2^* mutant, (2) *ninaE^P332^*, (3) *PBac cg17352^e01982^* (*culd^1^*), (4) *M9(vas-int.Dm)ZH-2A;M*(*3xP3-RFP.attP*)*ZH-86Fb*, (5) *arr1^1^ cn bw*, (6) *cn bw*, and (7) *w^1118^*. The *ninaE*-*trpl-gfp* flies were obtained from Dr Armin Huber (Department of Biosensorics, University of Hohenheim, Germany). The *ninaE-rh1-gfp* flies were maintained in the laboratory of T. W. Flies were maintained in 12-h-light–12-h-dark cycles with 2000 lux illumination at 25°C, except when mentioned differently in the text.

### Generation of transgenic flies

The *arr1*, *culd*, *rab5* and *rab7* cDNAs were amplified from the cDNA clones GH19095, GH11265, GH24702 and GH03685, respectively, and obtained from *Drosophila* Genomics Resource Center (DGRC). The *rab5 and rab7* cDNAs were cloned into the vector *pCDNA3-NRFP* (Invitrogen, Carlsbad, CA). The *arr1* cDNA was cloned into the vector *pIB-CBFP* (Invitrogen). The *culd* cDNA was cloned into the vectors *pCDNA3-CHA* and *pCDNA3-CRFP* (Invitrogen). The tagged cDNAs were amplified from these intermediary constructs and cloned into the expression vector *pninaE-attB*, which drives gene expression in R1–R6 photoreceptor cells (Wang and Montell, 2006). To express RFP under the control of the *culd* promoter region (*Pculd-rfp*), a 1.2-kb genomic DNA fragment (−1036 to +87 base pairs 5′ to the transcription start site) was cloned into the vector *pCDNA3-CRFP*, and the fragment including the *culd* promoter region and the *RFP* sequence was cloned into the vector *pUAST-attB*. These constructs were injected into *M(vas-int.Dm)ZH-2A;M(3xP3-RFP.attP)ZH-86Fb* embryos and transformants were identified on the basis of eye color (Bischof et al., 2007). The genomic region of *3xP3-RFP* was removed by crossing with *P(Crey)* flies.

### Generation of the *culd^2^* mutation

We produced the *culd^2^* mutation by using ends-out homologous recombination (Gong and Golic, 2003). The gene-targeting construct deleted a 617-bp sequence that included parts of the second and third exons (Fig. 1A). A 3.4-kb genomic fragment (extending from 3L 8348304 to 3L 8344885) and a 2.5-kb genomic fragment (3L 8351646 to 3L 8348921) were cloned into *pw35*. The gene-targeting construct was injected into *w^1118^* embryos, and transformants were identified on the basis of eye pigmentation. The final precise gene-targeted flies were screened by using eye pigmentation and genomic PCR. The *culd^2^* mutation was confirmed by using RT-PCR.

### Western blotting

For western blotting, fly heads were homogenized in SDS sample buffer with a pellet pestle (Fisher, Waltham, MA). Proteins were then fractionated by SDS-PAGE and transferred to Immobilon-P transfer membranes (Millipore, Bedford, MA) in Tris-glycine buffer. The blots were probed with primary antibodies against tubulin (mouse, 1:2000, Developmental Studies Hybridoma Bank), Rh1 (mouse, 1:2000 dilution, Developmental Studies Hybridoma Bank), GFP (rabbit, 1:2000, Torrey Pines Biolabs, Houston, TX), RFP (rabbit, 1:2000, Biovision, Milpitas, CA), TRPL (rabbit, 1:1000, Fisher, Waltham, MA), TRP (rabbit, 1:2000; Wang et al., 2008), INAD (rat, 1:2000; Wang et al., 2008), PDH (rabbit, 1:2000; Wang et al., 2010) and NORPA (rabbit, 1:2000; Wang et al., 2008). The TRP, INAD, PDH and NORPA antibodies were a gift from Dr. Craig Montell, Department of Molecular, Cellular & Developmental Biology, University of California, CA. Following probing with primary antibodies, the blots were incubated with IRDye 800 goat anti-rabbit-IgG, IRDye 680 goat anti-mouse-IgG, or IRDye 800 goat anti-rat-IgG antibodies (LI-COR Biosciences, Lincoln, NE). Signals were detected with the Odyssey infrared imaging system (LI-COR Biosciences). Blue light was filtered by using a Bandpass 10BPF10-480 filter (Newport, Irvine, CA) from a halogen lamp (Philips) operating at 150 W, and the light intensity delivered to flies was 34 mW/cm^2^. Orange light was filtered by a FSR-OG550 filter (Newport) from a halogen lamp (Philips) operating at 50 W, and the light intensity delivered to flies was 331 mW/cm^2^.

### Electroretinogram recordings

ERG recordings were performed as described previously (Wang et al., 2008). Two glass microelectrodes filled with Ringer's solution were inserted into small drops of electrode cream placed on the surfaces of the compound eye and the thorax. A Newport light projector (model 765) was used for stimulation. ERG signals were amplified with a Warner electrometer IE-210 and recorded with a MacLab/4s analog-to-digital converter and the clampelx 10.2 program (Warner Instruments, Hamden, CT). All recordings were carried out at room temperature.

### Subcellular fractionation

Twenty fly heads were collected and homogenized in lysis buffer (10 mM Tris-HCl pH 7.5, 150 mM NaCl, 0.5 mM EDTA) containing protease inhibitors (Roche). Homogenates were centrifuged at 950 ***g*** at 4°C for 10 min to remove debris. The supernatant was centrifuged at 21,000 ***g*** for 40 min at 4°C. The supernatant and pellet fractions were solubilized in SDS-loading buffer to equal volumes for western blot analysis.

### Immunohistochemistry

Retinas were dissected in phosphate-buffered saline (PBS; pH 7.4), fixed in 4% paraformaldehyde in PBS for 30 min and blocked in PBS plus 0.3% Triton X-100 (PBST) with 5% goat serum for 30 min. Retinas were incubated with primary antibodies against Rh1 (mouse monoclonal 4C5, 1:200, Developmental Studies Hybridoma Bank), GFP (rabbit, 1:200, Invitrogen), RFP (rat, 1:200, ChromoTek, Planegg, Germany) or PDH (rabbit, 1:200; from Dr. Craig Montell; Wang et al., 2010) in PBS plus 0.1% Triton X-100 (PBST) with 5% goat serum at 4°C overnight. Secondary antibodies against mouse, rabbit or rat IgG labeled with Alexa Fluor 488, Alexa Fluor 568 or Alexa Fluor 647 were used (1:500, Invitrogen). Samples were examined and images were recorded by using a Nikon A1-R confocal microscope and a Nikon eclipse Ni-U microscope (Nikon, Tokyo, Japan). Acquired images were processed by using Photoshop CS4 software and ImageJ. White-eyed flies were used in all immunohistochemistry experiments to avoid autofluorescence caused by eye pigmentation. No significant fluorescence was detected in any channels when the tissues were stained with secondary antibody only. The percentage of colocalization was calculated by using in the software Imaris X64 7.4.2 (Bitplane, Zurich, Switzerland). Briefly, protein-A-positive and protein-B-positive vesicles were marked by red and green dots, respectively. The total numbers of red, green and double-labeled dots were counted, and the corresponding percentages were calculated.

### Single ommatidia observation

Ommatidia from 3-day-old flies were dissected in Schneider's *Drosophila* medium (Invitrogen) as described previously (Xu et al., 2004). The images were taken within 30 min under a Nikon A1-R confocal microscope (Nikon, Tokyo, Japan). Colocalization percentages were calculated by using the software Imaris X64 7.4.2 as described above. Orange light (FSR-OG550, Newport) from a halogen lamp (operating at 50w) was used for illumination, and the light intensity delivered to flies was 331 mW/cm^2^.

### Transmission electron microscopy

To perform TEM, fly heads were dissected and prefixed in a solution with 4% paraformaldehyde and 2.5% glutaraldehyde for 2 h in ice, followed by fixation in 1% osmium tetroxide for 2 h at 4°C. Tissues were then dehydrated in a series of ethanol dilutions (10-min washes in 10, 25, 50, 75, and 100% ethanol), and embedded in LR white resin (Sigma, St Louis, MO). Thin sections (80 nm) prepared at a depth of 30–40 μm were stained with uranyl acetate and lead-citrate (Sigma), and examined by using a transmission electron microscope FEI Tecnai Spirit Twin (FEI, Hillsboro, OR).

### RNA-sequencing

Total RNA was purified by using Trizol reagent. The RNA integrity was checked by using a 2100 Bioanalyzer (Agilent Technologies, Santa Clara, CA) with a minimum RNA integrity number of 8. The mRNA was enriched by using oligo magnetic beads (Invitrogen) and fragmented to ∼150–250 bp. cDNAs were synthesized by using random hexamer primers and purified by using a MinElute PCR purification kit (Qiagen, Valencia, CA). The 42-cycle single-end sequencing was performed by using an Illumina Genome Analyzer IIx. CASAVA pipeline v1.8 was then used for sequence extraction and filtering. RNA-Seq reads were mapped to the fly genome by using the Tophat (v2.0.8b) software and the Ensembl genome annotation dataset (*Drosophila*_melanogaster.BDGP5.71.gtf). Then, the gene expression level fragments per kilobase of exon per million fragments mapped (FPKM) was estimated by using Cufflinks (v2.1.1) software.
